# Extracellular matrix remodeling fibroblasts govern the tumor microenvironment disparity between adenomatous lesions and adenocarcinoma in gallbladder

**DOI:** 10.3389/fimmu.2025.1637300

**Published:** 2025-07-18

**Authors:** Chuhan Ma, Huixin Hu, Hanrong Li, Bing Han, Chao Lv, Yu Tian

**Affiliations:** ^1^ Department of General Surgery, Shengjing Hospital of China Medical University, Shenyang, Liaoning, China; ^2^ Department of Gastroenterology, The First Affiliated Hospital of China Medical University, Shenyang, China

**Keywords:** tumor microenvironment, cancer-associated fibroblast, gallbladder cancer, gallbladder adenomatous lesions, collagen

## Abstract

**Introduction:**

Gallbladder cancer (GBC) is a highly lethal cancer with a poor prognosis. The adenoma-carcinoma sequence is a recognized model for GBC development, but its underlying mechanisms are not well understood.

**Methods:**

Human specimens were collected from Shengjing Hospital of China Medical University. Single-cell isolation and sequencing were conducted on cells from four GBC and four gallbladder adenomatous lesions (GBA) samples, and the raw gene expression matrices were analyzed using R software with the Seurat package. This included cell type annotation, differential gene expression analysis, functional enrichment, and gene set score calculation. Additional analyses such as protein-protein interaction network, immune infiltrate analysis, high-dimensional weighted gene co-expression network analysis, and cell-cell communication analysis were also performed.

**Results:**

The study revealed that epithelial-mesenchymal transition (EMT) plays a key role in the malignant transformation of epithelial cells from GBA to GBC. The immune landscape of GBC is predominantly immunosuppressive compared to the inflammatory environment within GBA. A specific subset of fibroblasts with extracellular matrix remodeling capabilities appears to be a major driver of the TME differences between GBC and GBA, potentially acting through COL1A2-mediated cell communication.

**Discussion:**

This work highlights the distinct roles of various cell types in the TME of GBA and GBC, and emphasizes the importance of understanding the mechanisms of malignant transformation from adenomatous lesion to carcinoma in the gallbladder. The findings pave the way for further research into the mechanisms underlying the adenoma-carcinoma sequence.

## Introduction

Gallbladder cancer (GBC) is a relatively infrequent but highly lethal cancer with a poor prognosis. Surgical resection is the most promising therapeutic approach to achieve a complete cure in the current clinical landscape (1, 2). However, only 30% of GBC patients are diagnosed or suspected preoperatively, mainly owing to the absence of specific symptoms of early-stage disease (3). Most symptomatic GBC patients have an incurable tumor and the recurrence rate in resected GBC is disproportionately high, with a 5-year relative survival rate of only 28% in regional disease (3). However, the susceptible population of GBC including patients with gallbladder inflammation or adenomatous lesions often have a good prognosis (4). Therefore, it is critical to study the tumorigenesis mechanism in the gallbladder to identify high-risk patients with precancerous lesions of GBC.

Cholelithiasis is one of the major risk factors for gallbladder cancer (4). Typically, it is asymptomatic but has the potential to trigger recurrent inflammation, which can damage the epithelial cells. Repeated injury, repair, and regeneration of the gallbladder mucosal epithelium lead to precancerous states for GBC such as metaplasia and dysplasia (3, 4). Following the adenoma-carcinoma sequence, GBC can also develop from adenoma precursor lesions, including intracholecystic papillary neoplasm (ICPN) and pyloric gland adenoma. Most adenomatous lesions occur in the context of non-lithiasic inflammation and the ICPN is recognized as one of the precursor lesions to gallbladder cancer (3). However, it remains a challenge to understand the mechanism of malignant transformation of gallbladder adenomatous lesions (GBA).

Chronic inflammation contributes to the development of both GBC and GBA (3). However, they exhibit distinct biological behaviors and patients’ prognoses. Cancer is a complex ecosystem with a wide range of cells including epithelial cells, immune cells, stroma cells, and other types, such as adipocytes and neurons. Both the accumulation of mutations in epithelial cells and alterations in the tumor microenvironment contribute to tumorigenesis (5). During the process of tumorigenesis, tumor microenvironment (TME) tips the balance from immune surveillance to immune evasions as a consequence of the decrease in cytotoxic CD8^+^T and NK cells, increased immunosuppressive regulatory T cells (Tregs), exhausted CD8^+^T cells, and helper T lymphocyte type 2 (Th2) polarization. In parallel, tumor-promoting myeloid cells, such as M2 macrophages and myeloid-derived suppressor cells (MDSCs) also accumulate in TME, and DCs display defective antigen presentation ability (5). ECM remodeling mediated by stroma cells, especially cancer-associated fibroblasts (CAFs), also plays an important role in tumorigenesis (6). Deciphering the TME difference between GBA and GBC provides an appropriate model to compare the chronic inflammation that facilitates tumorigenesis and cancer-associated inflammation, and contributes to revealing the mechanisms of GBA malignant transformation and poor prognosis of GBC.

In this study, we used single-cell RNA sequence (scRNA-seq) analysis to compare the TME difference between GBC and GBA and find that a type of tumor-specific CAFs can interact with most cell subsets enriched in GBC to facilitate the formation of TME. This study paves the way for further research on the deep mechanisms underlying the adenoma-carcinoma sequence.

## Materials and methods

### Human specimens

We collected 4 GBC samples (Pathological diagnosis: gallbladder adenocarcinoma) and 4 GBA samples (Pathological diagnosis: ICPN) for this study at Shengjing Hospital of China Medical University, Shenyang, China. Patients with radiologic evidence suggestive of distant metastasis or a history of any pre-operative physical or pharmacological therapy were excluded from the study. At least three pathologists confirmed the patients’ histological diagnoses of gallbladder disease (**Supplementary Data 1**).

### Single-cell Isolation and sequencing

The fresh tissue specimens were minced and subsequently digested with 0.25% trypsin. The resulting cell suspension was filtered through a 40μm sterile strainer. Next, add the red blood cell lysis buffer to the cell suspension. This mixture was incubated at room temperature for 2-5 min. To remove the supernatant, the mixture underwent centrifugation at 300g at 4°C for 5 min following incubation, and the remaining pellet was gently suspended in PBS. After cell counting and viability assessment, the prepared cell suspension (cell viability>85%, 500-1500 cells/μl) was subjected to microfluidic chip-based encapsulation, where magnetic beads tagged with barcodes and cells were encapsulated within droplets. Following the collection of oil-in-water emulsion droplets, cell lysis was performed and reverse transcription was carried out within the droplets. Finally, the emulsion was broken, and a complementary DNA library was constructed. Sequencing was conducted on the DNBSEQ platform.

### Single-cell RNA-seq data processing

We got the raw gene expression matrices for each sample through the Cell Ranger Pipeline and analyzed the output-filtered gene expression matrices using R software (version 4.3.1) with the Seurat package. Cells with expression of more than 200 genes were selected for further analyses. Low-quality cells were omitted according to the following criteria: (1) <200 or > 7000 genes; (2) >20% unique molecular identifiers (UMIs) derived from the mitochondrial genome. After the removal of low-quality cells, we normalized the gene expression matrices, identified 2000 features with high cell-to-cell variation, and reduced the dimensionality of the datasets through the principal component analysis (PCA) on linear-transformation scaled data. The harmony algorithm was employed to correct batch effects in our single-cell sequencing data. Finally, we clustered cells using the FindNeighbors (top 50 PCs) and FindClusters functions. After that, we performed non-linear dimensional reduction with the RunTSNE function. Based on the above analysis, we clustered each cell subtype with the same process.

### Cell Type annotation and cluster markers identification

After non-linear dimensional reduction and projection of all cells into two-dimensional spaces by t-distributed stochastic neighbor embedding (t-SNE), cells clustered together according to common features. We classified and annotated cell clusters based on the expression of canonical markers or recorded gene signatures of particular cell types.

Considering the inevitable bias in data processing and the impact of trypsin-based dissociation on cellular gene expression, we excluded cells with high expression of mitochondrial and stress-related genes when identifying cell subtypes.

### DEG identification, functional enrichment, and gene set score

Differential gene expression analysis was performed using the GEO2R in bulk RNA sequence (bulk RNA-seq) of GBA and GBC. Differentially expressed genes (DEGs) were filtered according to a minimum log fold change (logFC) of 1 and a maximum adjusted *p* value of 0.05. For scRNA-seq, differential gene expression analysis was performed using the FindMarkers function in Seurat. DEGs were filtered according to a minimum logFC of 0.25 and a maximum adjusted *p* value of 0.05.

We conducted enrichment analysis for the functions of the DEGs using metascape and clusterprofiler package based on Gene Ontology (GO), Kyoto Encyclopedia of Genes and Genomes (KEGG) and Hallmark database. We also used the Ucell package to calculate the gene set score on each cell.

### Protein-protein interaction network

We input all DEGs between GBC and GBA in the GSE202479 dataset into the STRING database (string-db.org) and set the minimum required interaction score as 0.7 (high confidence). Subsequently, we calculated the Betweenness centrality (BC) to evaluate the importance of the protein through cytoNCA and visualize the PPI network using Cytoscape. 50 genes with the highest BC values were exported to construct a PPI network, with the top 10 genes being regarded as hub genes and placed at the center of the network.

### Immune infiltrate analysis

We used CIBERSORT method to calculate the proportion of 20 types of immune cells based on transcriptional data of the GSE202479 dataset and calculated immune and stroma cell infiltration scores through ESTIMATE. All parameters were assigned their default values for the execution of immune infiltration analysis.

### High dimensional weighted gene co-expression network analysis

We used hdWGCNA package to construct metacells for each sample and each cell cluster. To establish co-expression networks, we applied a soft threshold power to distinguish modules with distinct expression patterns. The optimal soft threshold power was determined using the ‘pickSoftThreshold’ function. Our samples were classified into three distinct pathological phenotypes: GBA, early GBC (confined to the gallbladder, T1-2), and advanced GBC (T3). Subsequently, we performed the standard hdWGCNA pipeline and calculated the correlations between modules and pathological phenotypes.

### CytoTRACE analysis

We used the CytoTRACE package to calculate the CytoTRACE score of epithelial cells. The CytoTRACE score reflects cellular stemness: higher scores denote greater stemness and less differentiation, whereas lower scores indicate the opposite.

### CNV analysis

We used the inferCNV to infer copy number variations (CNVs) in each chromosomal region of the tumor genome compared to normal cells. We designate all epithelial cells and endothelial cells from the same sample as an interrogation group. Endothelial cells from each patient were considered as reference and spike-in. All samples were analyzed following the same process.

### Cell trajectory analysis

We used Monocle2 packages to construct trajectories to discover the fibroblast and epithelial transitions. The process began with single-cell RNA sequencing data, monocle dataset construction, size factor, and dispersion estimation. We screened genes with expression over 0.1 and dispersion over 1. These filtered genes were sorted through DDRTree for dimensionality reduction. The resulting cell trajectories were graphically represented and assigned colors based on Seurat clustering, states, tissue types, and pseudotime. Cell trajectory analysis also revealed changes in gene expression across pseudotime. Branched Expression Analysis Modeling (BEAM), a statistical approach integrated within the Monocle2 package, is designed to identify genes that display distinct expression profiles corresponding to trajectories as cells commit to divergent fates throughout developmental processes or disease progression. We identified BEAM genes at each branch point of the trajectories with q<0.01. VECTOR package was also used to identify the starting cells and infer the vectors of developmental directions for T cells in uniform manifold approximation and projection (UMAP).

### Transcription factor prediction

We used SCENIC to perform transcription factor (TF) prediction. We used GENIE3 to obtain the co-expression modules and downloaded the motifs database for Homo sapiens from the website (https://resources.aertslab.org/cistarget/databases/). The input matrix was the normalized expression matrix of mesenchymal cells.

### Cell-cell communication analysis

We used CellChat package to assess cell-cell interactions between different cell types in the dataset. CellChat model the probability of cell–cell communication by integrating gene expression with the CellChatDB.human database. We analyzed cell-cell interactions separately under different conditions following the default pipeline. Normalized count data from each condition were used to create CellChat objects, and recommended preprocessing functions were applied to analyze individual datasets with default parameters. All ligand-receptor interaction categories in the database were used in the analysis. Ligand-receptor pairs were filtered based on a threshold of *p*<0.01.

### Immunohistochemistry

For multiplex immunohistochemistry (mIHC), we used xylene and alcohol to dewax and rehydrate slides, followed by antigen retrieval in ethylenediaminetetraacetic acid (EDTA) via high pressure for 1.5 minutes. The slides were immersed in a 3% hydrogen peroxide solution to inactivate endogenous peroxidases. After blocking the slides with a 3% bovine serum albumin (BSA) solution for 30 minutes, the staining procedure was initiated. We added the primary and secondary antibodies followed by the direct application of 50ul TYR-520 fluorescent dye, TSA fluorescent dye reagent, and 0.1μ enhancer. After washing with PBS, repeated the antigen retrieval and staining steps with a different primary antibody and fluorescent dye (TYR-570). DAPI was utilized for nuclear counterstaining, and an anti-fade mounting medium was employed to seal the slides. Slides were then examined and imaged using a confocal microscopy system and the expression of various markers being assessed through the ScanViewer software.

Paraffin-embedded tissue was cut into 4-μm sections and mounted on glass slides. After dewaxing and rehydration, antigen retrieval was performed. Sections were then washed with PBS, blocked with 10% normal goat serum for 10 min, and incubated with mouse monoclonal antibody (anti-Twist1, 1:100; anti-SNAI1, 1:50) at room temperature for 1 hour. Following primary antibody incubation, sections were incubated with a biotinylated goat anti-mouse secondary antibody at room temperature for 10 min. After rinsing with PBS, sections were incubated with streptavidin–biotin–peroxidase complex at 24–27°C for 10 min and stained with DAB until brown. Finally, slides were dehydrated, cleared, and coverslipped for observation. The immunohistochemical staining is scored based on two aspects. For cell - staining intensity, it is divided into 4 levels: 0 points for no positive staining (negative), 1 point for weak staining, 2 points for moderate staining, and 3 points for strong staining. For the percentage of positive cells, it is also graded into 4 levels: 1 point for ≤25%, 2 points for 26% - 50%, 3 points for 51% - 75%, and 4 points for >75%. The final score is obtained by multiplying the score of cell-staining intensity by the score of the percentage of positive cells.

### Statistics

We utilized R software for the statistical analysis of single-cell RNA sequencing data. Based on the central limit theorem, a t-test was applied to compare the gene set enrichment and CNV scores between the two groups. The Pearson correlation test was also employed to determine the correlation between epithelial-mesenchymal transition (EMT) and CNV scores.

## Results

### Mesenchymal cells determine the TME difference between GBC and GBA through collagen secretion

**Figure 1:**
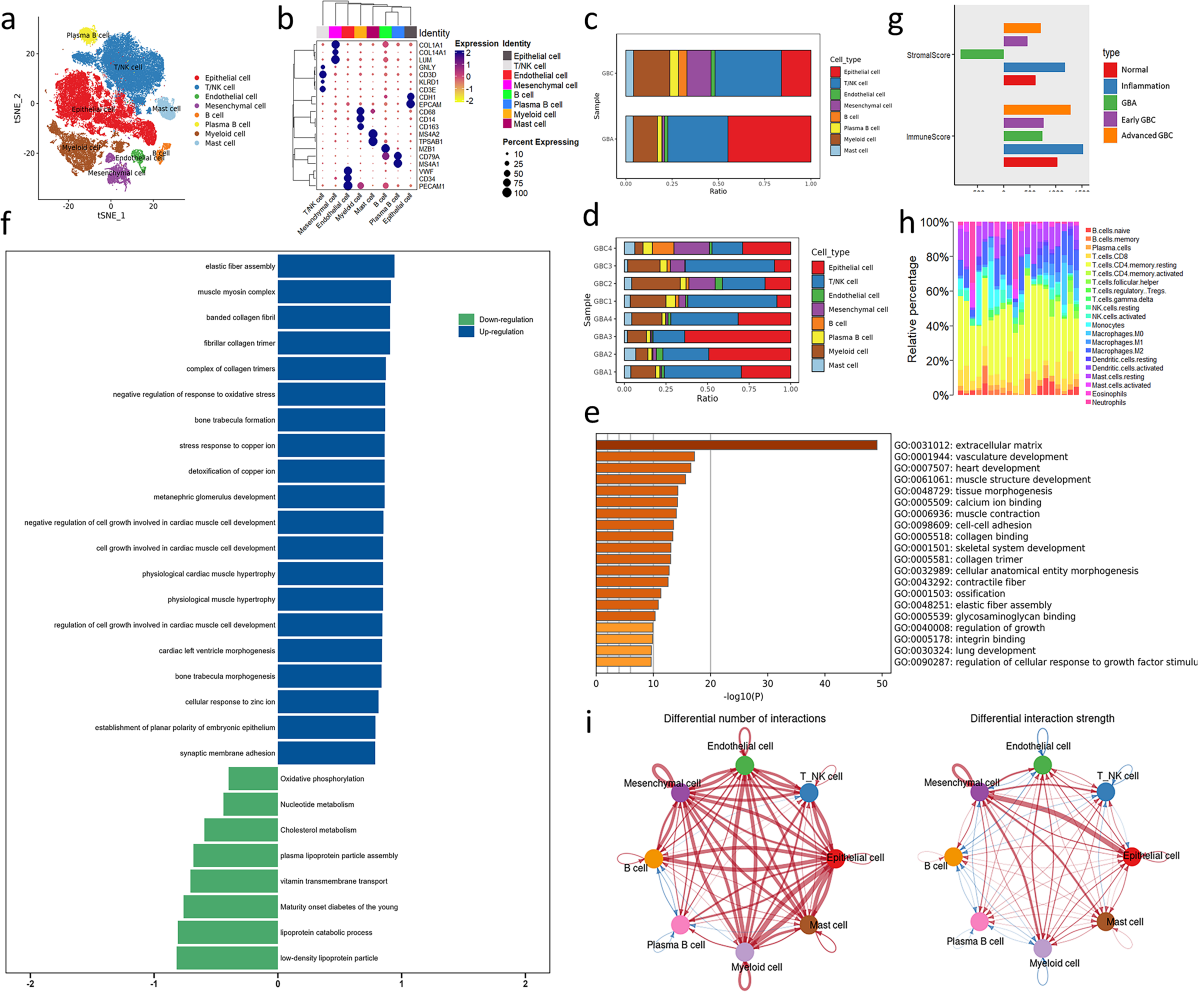
Overview of bioinformatics analysis across GBC and GBA. **(a)** t-SNE plot visualizing 8 cell types from 8 samples. **(b)** Dotplot plot visualizing expression levels of cell-type gene signatures among identified cell types. **(c, d)** Bar charts showing the relative abundance of various cell types in GBC and GBA and various cell types in each sample. **(e, f)** GO and GSEA enrichment analysis for the DEGs between GBC and GBA in GSE202479. **(g)** Stroma and immune cell infiltration score of samples with different gallbladder disease in GSE202479. **(h)** Immune cells infiltration prediction of GBC and GBA samples in GSE202479. **(i)** Differential number and strength of intercellular interactions between GBC and GBA. Red represents upregulated intensity in GBC, while blue represents downregulated intensity; the width of line is the extent of upregulation or downregulation. .

To profile the TME landscape of gallbladder-derived neoplasm, we used the droplet-based scRNA-seq platform to compile a single-cell atlas from 8 surgically resected fresh tissues, including 4 primary gallbladder adenocarcinomas and 4 ICPNs. Following resection, digestion, and quality filtering, we obtained 70,041 cells. We identified 8 cell subpopulations using t-SNE method, including T/NK cells (n=23,574; CD3D, CD3E,GNLY,KLRD1), B cells (n = 1,220;CD79A, MS4A1), plasma B cells (n = 2,238; CD79A,MZB1), myeloid cells (n= 10,761; CD68, CD14, CD163),mast cells (n= 2.813; TPSAB1,MS4A2), mesenchymal cells (n= 3,730; COL1A1, COL14A1, LUM), endothelial cells (n= 1.361; CD34, PECAM1, VWF), and epithelial cells (EPCs, n= 24,344; EPCAM, CDH1) (**Figures 1 a, b**; **Supplementary Figure S1**). To investigate the difference in TME between benign and malignant tumors of the gallbladder, we calculated the cell ratio of GBA and GBC. Compared with GBA, more mesenchymal cells, myeloid cells, and B cells exist in GBC’s TME (**Figures 1c, d**). Notably, the epithelial cells occupy a smaller proportion in GBC than GBA, which is consistent with the higher proliferative score of GBA epithelial cells (**Supplementary Figure S2a**). That indicates the pivotal role of non-cancerous cells in the poor prognosis of GBC.

We also performed differentially expressed gene analysis between bulk-RNA seq of 10 GBC and 3 GBA samples from the GSE202479 dataset and constructed a protein-protein interaction (PPI) network of these DEGs (**Supplementary Figure S3**). Many hub genes in the PPI network are components of the extracellular matrix (ECM), such as COL1A2, COL3A1 and FBN1.GO and GSEA analysis shows that the DEGs are significantly enriched in terms associated with stroma components of TME, such as ECM, vasculature development, and elastic fiber assembly (**Figures 1e, f**). The stroma cell infiltration score of GBC is also obviously higher than GBA in this dataset (**Figure 1g**). We also predicted the immune cell infiltration of these samples using CIBERSORT. The results show that the proportion of immune cells varies greatly between different samples. However, GBC samples always contain a higher abundance of myeloid cells than GBA in this dataset (**Figure 1h**).

Cellchat analysis revealed that the number and strength of cell interaction is upregulated significantly, and mesenchymal cell is the center of this difference, especially in epithelial cells. That indicates that mesenchymal cells determine the difference in TME between GBC and GBA (**Figure 1i**). Among all the interactions mediated by mesenchymal cells, the COLLAGEN signaling pathway caught our interest among all the interactions due to its high communication probability (**Supplementary Data 2**). We noticed that the most upregulated interaction of mesenchymal cells with epithelial cells is COL1A2-SDC1. In immune cells, including T/NK cells, myeloid cells, and mast cells, the interaction is mostly upregulated in collagen-CD44 (**Supplementary Figure S4**). In the following sections, we will detail the TME difference between GBC and GBA and clarify how the mesenchymal subtype dominates this difference. Our focus will be on collagen signaling and the cell subpopulations interacting with unique mesenchymal cell subtypes.

### ECM-remodeling fibroblasts dominate the collagen-mediated cell interaction in the TME of GBC

**Figure 2:**
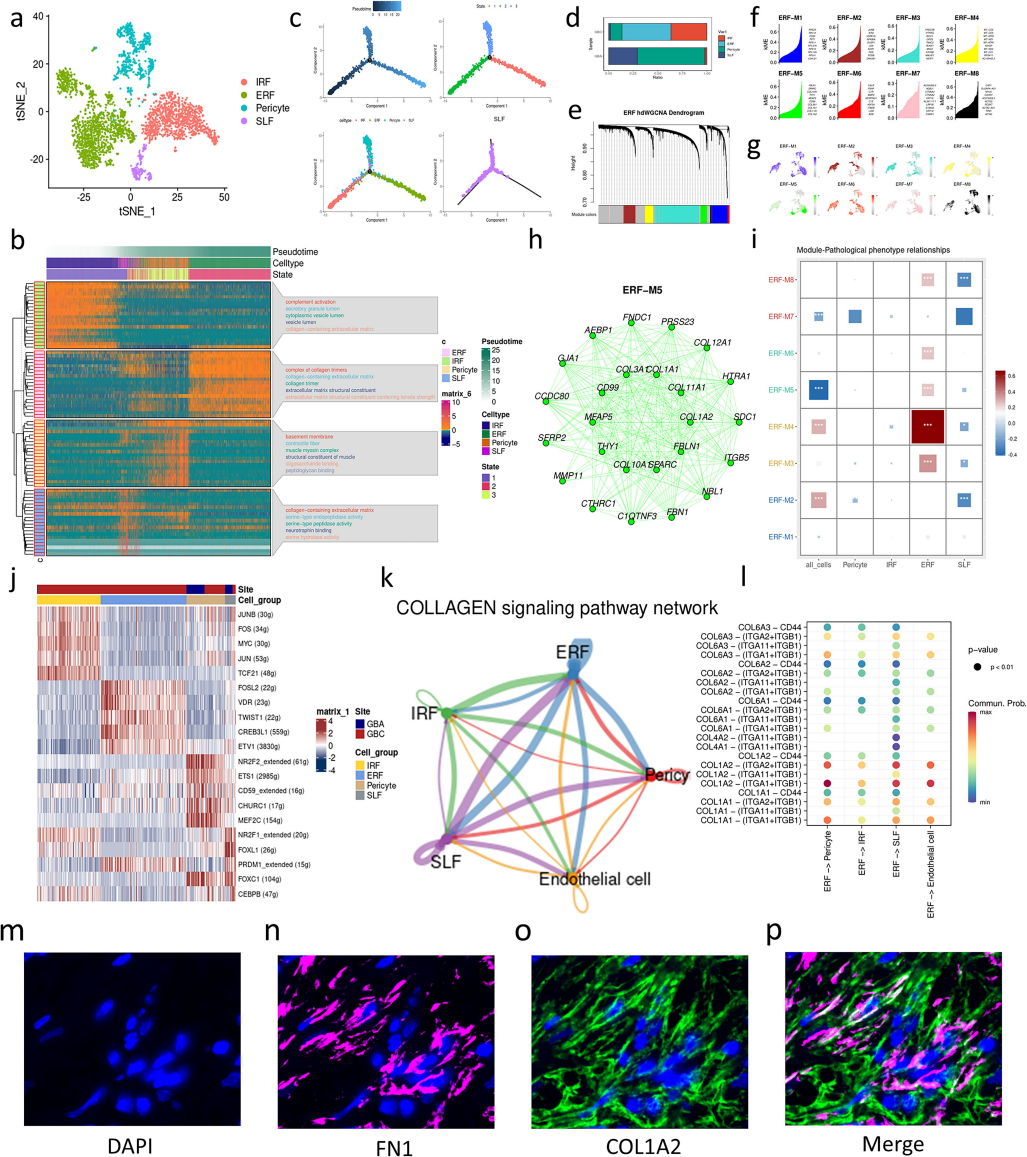
Characterization of mesenchymal cell subtypes in GBC and GBA. **(a)** t-SNE plot visualizing mesenchymal cell subsets from GBC and GBA. **(b)** Enrichment analysis and the change over pseudotime in gene markers of mesenchymal cell subtypes. **(c)** Cell trajectory of mesenchymal cell subtypes. **(d)** Bar charts showing the relative abundance of various cell types in GBC and GBA and various cell types in each sample. **(e)** Dendrogram of 8 modules in the scale-free network. **(f)** Hub genes of each gene modules of ERF. **(g)** Projection of gene expression in each module on umap plot. **(h)** Top 25 Genes with the highest eigengene-based connectivity (kME) in ERF-M5. **(i)** The correlation between modules and clinical traits. The area and color of squares represent the correlation; *(p<0.05), ** (p<0.01), *** (p<0.001). **(j)** Gene expression heatmap for TFs of each mesenchymal cell subtypes. **(k)** Cell-cell interaction of stroma cells. **(l)** Dotplot shows the cell communication between ERFs and other stroma cells through collagen signaling. The colors represent cell types and the width of line represents the intensity of cell communication. **(m-p)** mIHC images displaying the expression of ERF markers (FN1, COL1A2) in GBC tissue.

To identify the fibroblast subpopulation mediating collagen signaling, we reclustered the mesenchymal cells and identified four cell subtypes according to the markers provided by Wang, et al (7), including immunity-regulating fibroblasts (IRF), ECM-remodeling fibroblasts (ERF), senescence-like fibroblasts (SLF) and pericytes (**Figure 2a**). GO enrichment analysis reveals that IRFs are engaged in complement activation and vesicle secretion (**Figure 2b**). ERFs highly express genes associated with collagen biosynthesis and are enriched in GO terms about ECM remodeling (**Figure 2b**). SLF is characterized by the secretory capacity of senescence-associated secretory phenotype (SASP) with high expression of protease or protease inhibitors (CST1, PAPPA, CTSC, PLAT) (**Supplementary Figure S2b**). The gene markers of SLFs are enriched in the degradation of serine (**Figure 2b**), which is a manifestation of cell senescence. Pericyte is a type of mesenchymal cells that surround the endothelium of capillaries. Notably, the pericytes in our data are a collection of classic pericytes and vascular smooth muscle cells (VSMCs) due to the limitation of cell number and massive gene overlaps between pericytes and VSMCs (**Supplementary Figure S2b**). Consistent with previous research, pericytes are scarce in tumors, which indicates that vessels in GBC are poorly covered by pericytes (8). To depict the transition between these mesenchymal cells, we performed an unsupervised trajectory analysis and found that IRFs, ERFs, and pericytes represent three different states in the cell trajectory. Notably, SLFs cross three states of the cell trajectory (**Figure 2c**). Therefore, SLFs might be the common destination of these mesenchymal cells, considering their high expression of senescence-related genes (7).

ERF is the main source of collagen of ECM (**Supplementary Figure S2b**), and they are enriched in GBC but rare in para-carcinoma tissues and GBA (**Figure 2d**) (**Supplementary Figures S2c-e**). To further investigate the functions of ERFs, we performed high dimensional weighted gene co-expression network analysis (hdWGCNA) using 7 as soft power threshold and identified eight gene modules of ERFs (**Figures 2e, f**) (**Supplementary Figure S2f**). We divided our samples into three pathological phenotypes including GBA, early GBC (confined to the gallbladder, T1-2), and advanced GBC (T3). After that, we calculated the correlations between modules and pathological phenotypes. M3, M4, M5, M6, and M8 exhibit significant positive correlation with pathological phenotypes, especially M4 (**Figure 2i**). M4 contains many mitochondrial genes associated with oxidative stress (**Figure 2f**). However, M5 attracted our interest due to its specific expression in ERFs (**Figure 2g** **) (** **Supplementary Figure S2g**). M5 contains genes associated with ECM remodeling, especially different types of collagens (**Figure 2h**). These results also support that ERF is associated with the progression of GBC and play an important role in ECM remodeling through collagen secretion.

To explore the origins of ERFs, we also performed BEAM analysis and TF prediction using SCENIC. In the model of Wang, et al. (7), IRFs can transform into ERFs. Our study revealed the up-regulation of genes associated with ECM degradation and collagen synthesis during the IRFs transition to ERFs (**Supplementary Figure S2h**), which is consistent with the change of ECM components in cancer progression (9). FOSL2, VDR, TWIST1, CREB3L1, and ETV1 are predicted to be the TFs of ERFs (**Figure 2j**). TWIST1 and CREB3L1 are associated with tumor progression mediated by CAF-induced ECM remodeling and fibrosis (10, 11). JUN, FOS, and JUNB are TFs of IRF, which are members of the AP-1 family and can drive CAF activation (12) (**Figure 2j**). FOSL2, TF of ERF, is also a member of the AP-1 family, implying the correlation between ERF and IRF. Cellchat analysis reveals that ERFs can interact with pericytes and endothelial cells through COL1A2-(ITGA1+ITGB1) and COL1A2-(ITGA2+ITGB1) (**Figures 2k, l**), which is consistent with the increased cell interaction in GBC among mesenchymal cells subtypes and between mesenchymal cells and endothelial cells. The result also supports that ERFs play a dominant role in the increased collagen-mediated cell interaction between GBC and GBA. We performed mIHC on GBC tissue paraffin section using the ERF markers FN1 (IHC score=2) and COL1A2 (IHC score=6). The results revealed that ERFs are enriched in GBC tissues and surrounded by secreted COL1A2 protein, which supports that ERF can secrete COL1A2 to interact with other cells in GBC (**Figures 2m–p**).

### Epithelial-mesenchymal transition contributes to the malignant transformation of GBA

**Figure 3:**
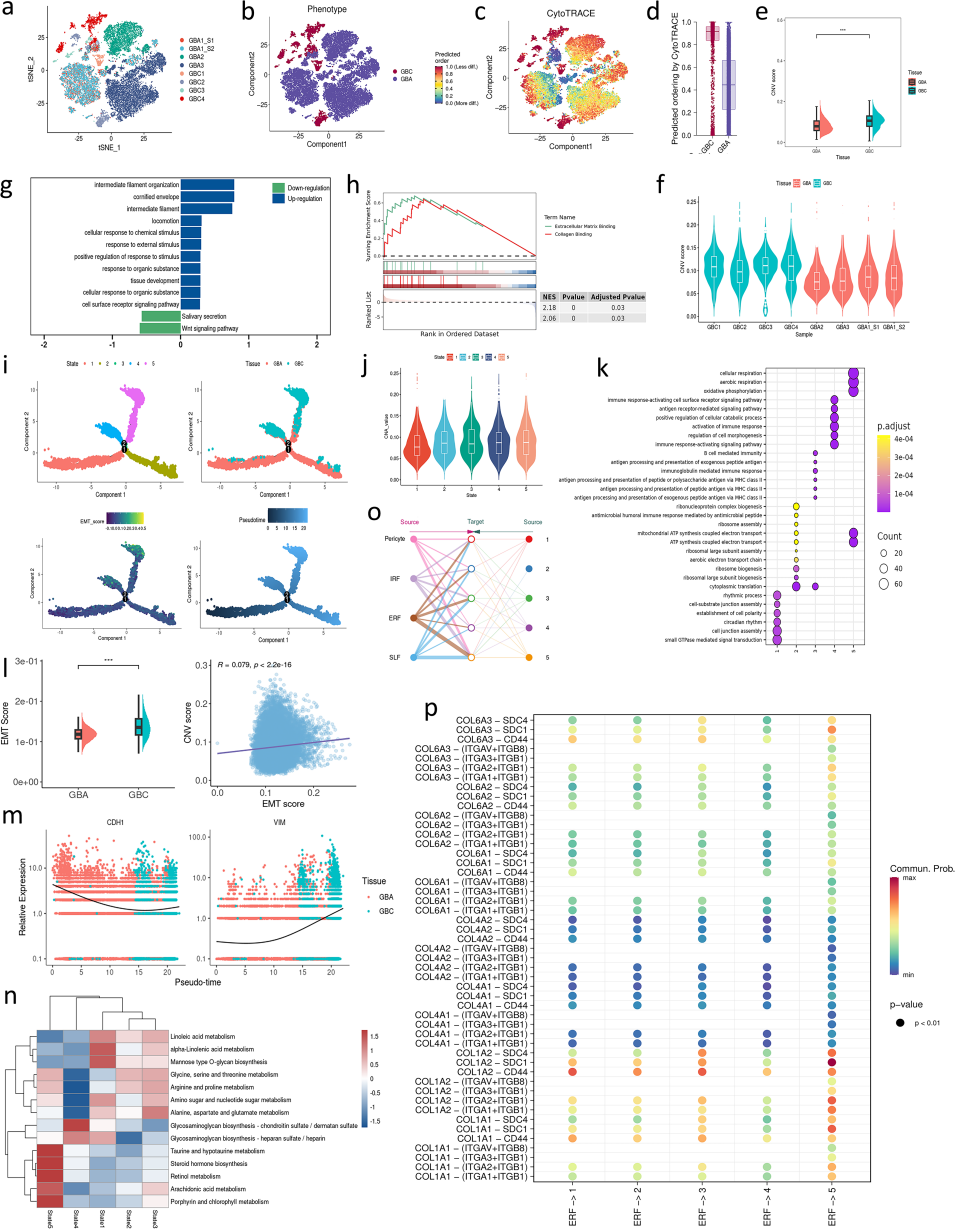
**(a)** t-SNE plot visualizing epithelial cells colored by samples. **(b)** t-SNE plot visualizing epithelial cells colored by tissue type. **(c)** Projection of CytoTRACE scores in each module on t-SNE plot. **(d)** CytoTRACE scores for epithelial cells in GBA and GBC. **(e, f)** CNV scores for epithelial cells in GBA and GBC. **(g, h)** GSEA enrichment analysis for the DEGs of epithelial cells between GBC and GBA. **(i)** Cell trajectory of mesenchymal cell subtypes. **(j)** CNV scores in each state of epithelial cells. **(k)** enrichment analysis for the DEGs of epithelial cells between GBC and GBA. **(l)** CNV scores and their correlation with CNV scores for epithelial cells in GBA and GBC. **(m)** The dynamic expression of CDH1 and VIM in a pseudotime. **(n)** The metabolism of epithelial cells in each state. **(o)** The cell communication between each state of epithelial cells and mesenchymal cell subtypes. **(p)** Dotplot shows the cell communication between ERFs and each state of epithelial cells through collagen signaling.

To investigate the genetic difference between GBC and GBA, we projected the epithelial cells on a t-SNE plot. These cells are well separated according to samples or tissue type (**Figures 3a, b**). In addition, the epithelial cells of two samples from one patient are incorporated into the same cell clusters (**Figure 3a**), indicating that the separation of cell clusters is attributed to the high genetic heterogeneity of gallbladder neoplasm instead of batch effect. We also calculated the CytoTRACE score and CNV score of each epithelial cell. As we expected, both CytoTRACE and CNV scores of GBC are significantly higher than GBA, indicating higher stemness and genetic variation of tumor cells (**Figures 3–f**). GSEA analysis of DEGs between GBC and GBA revealed that the malignant cells upregulate the response to external stimulus and the ability to interact with ECM (**Figures 3g, h**). GBA upregulates Wnt signaling (**Figure 3g**), which is consistent with previous research (13). Wnt signaling plays an important role in EMT, a process by which epithelial cells lose polarity and acquire invasiveness (14), implying that EMT might take part in the malignant transformation of GBA.

To depict the malignant transformation between GBA and GBC, we performed unsupervised trajectory analysis using monocle2. The cell trajectory reveals the evolution from GBA to GBC following pseudotime (**Figure 3i**). In this trajectory, state 1 represents the epithelial cell with the potential to differentiate into adenoma or cancer cells. The marker genes of state 1 are enriched in establishing cell polarity and the cell junction assembly, exhibiting the loss of epithelial signatures. State 2 is mainly composed of adenoma cells, with high expression of genes associated with protein synthesis, indicating their high proliferative ability. State 3 is a turning point in the fate of adenoma cells, after which they will develop into two different types of cancer cells. Cancer cells in state 4 exhibit high regulatory activity to activate the immune response, while those in state 5 exhibit the enhanced ability of oxidative phosphorylation (**Figure 3k**). The CNV scores of state 4 and state 5 are significantly higher than those of state 1, 2, and 3 (**Figure 3j**). ScMetabolism shows state1, 2 and 3 mainly involve amino and fatty acid metabolism. State 4 exhibits significant biosynthetic capacity of glycosaminoglycan, a component of ECM. State 5 mainly takes part in the metabolism of lipid mediators with bioactivity, such as arachidonic acid and steroid hormones (**Figure 3n**). We also performed BEAM analysis to detect the gene variation during the evolution from GBA to GBC. Through enrichment analysis, we noticed that EMT is enriched in BEAM genes of each branch point, which supports that EMT plays an important role in the malignant transformation of GBA (**Supplementary Figures S5a, b**). To confirm our hypothesis, we calculated the EMT score of each epithelial cell using UCell and found that the EMT score of GBC was significantly higher than that of GBA (**Figure 3l**). Besides, the EMT score increases along with the cell trajectory (**Figure 3i**). The mesenchymal marker VIM (Vimentin) also increases while the epithelial marker CDH1 (E-cadherin) decreases over pseudotime (**Figure 3m**). IHC demonstrated higher expression of the EMT markers Snail (GBC: IHC score=8; GBA: IHC score=4) and Twist (GBC: IHC score=6; GBA: IHC score=3) in GBC tissues than in GBA (**Supplementary Figure S5c**). CNV score is a widely -used method to estimate the malignancy of epithelial cells. EMT scores positively correlate with CNV scores in our data (**Figure 3l**). The DEGs between early GBC and GBA in the GSE202479 dataset are also enriched in EMT (**Supplementary Figure S5d**), which also demonstrates that EMT contributes to the malignant transformation of GBA.

ERFs exhibit a wide range of interactions with epithelial cells. Cellchat analysis revealed that the increased cell interaction in GBC is attributed to the COL1A2-SDC1 between ERFs and state 5. However, state 4 exhibits weak interaction with ERFs (**Figures 3o, p**). Our analysis suggests that ERFs have increased interactions with tumor cells at later stages (higher CNV score) characterized by aberrant metabolic profiles, rather than those actively participating in ECM formation. According to previous studies (15, 16), SDC1 is essential for Wnt-1 induced mammary tumorigenesis, which implies that COL1A2-SDC1 interaction might facilitate the Wnt-induced malignant transformation of GBA cells.

### Identification of myeloid lineage that communicates with ERFs

As we mentioned, mesenchymal cells mainly interact with immune cells through collagen-CD44. CD44 is a kind of adhesion molecule and takes part in the tissue retention of immune cells, which facilitates the formation of TME. In the following sections, we will discuss the features of immune cells that interact with ERFs in detail.

Tumor-infiltrating myeloid cells are composed of monocytes, macrophages, DCs, and neutrophils. Due to the limitations of the C4 and 10x platforms, neutrophils are absent in our data. Therefore, we divided the myeloid cells into two groups: monocytes/macrophages and DCs according to canonical markers.

**Figure 4:**
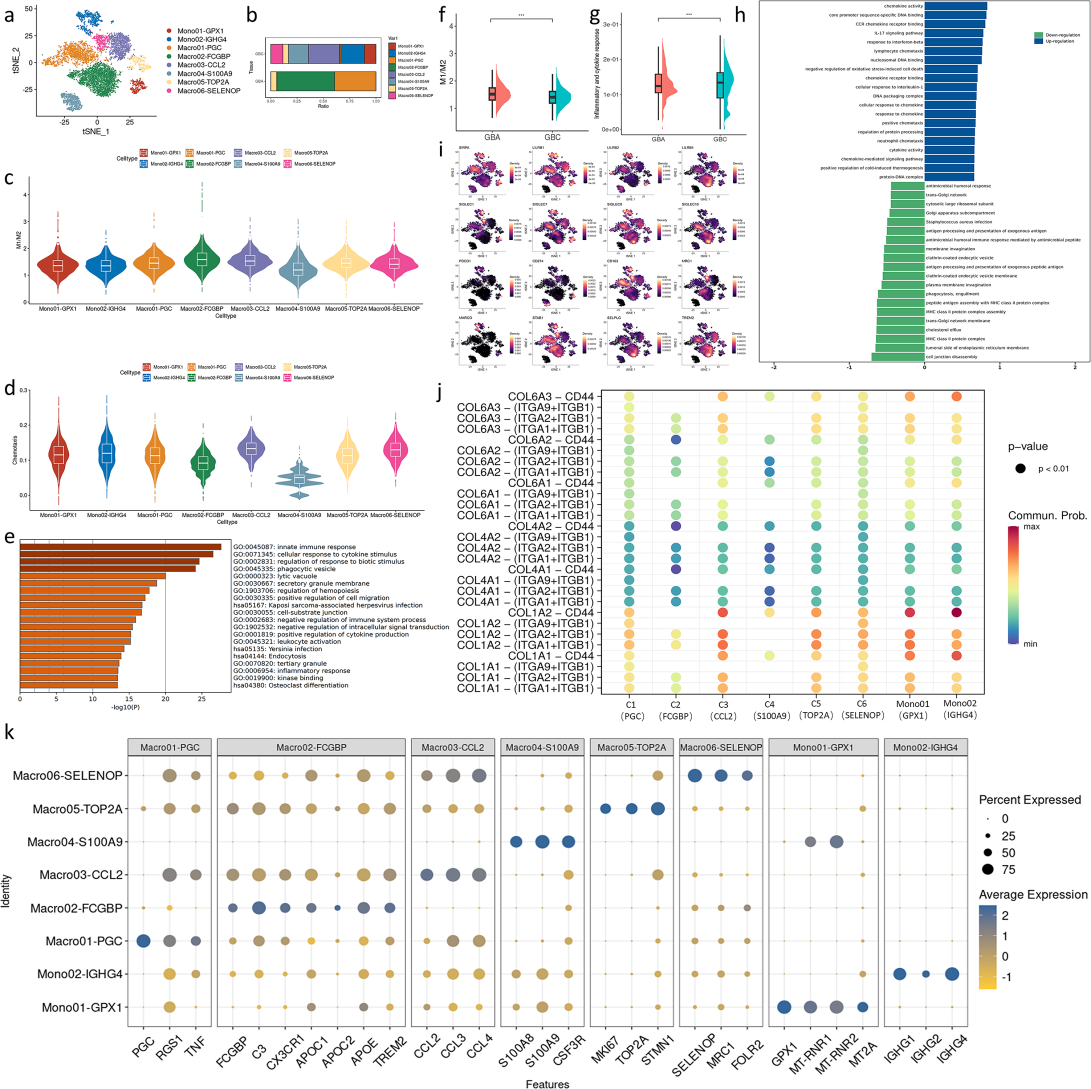
**(a)** t-SNE plot visualizing macrophage subsets from GBC and GBA. **(b)** Bar charts showing the relative abundance of various macrophages subtypes in GBC and GBA. **(c)** M1/M2 scores in macrophage subtypes. **(d)** Chemotaxis scores in macrophage subtypes. **(e)** GO enrichment analysis of markers of Macro04. **(f)** M1/M2 scores in macrophages between GBC and GBA. **(g)** M1/M2 scores in macrophages between GBC and GBA. **(h)** GSEA enrichment analysis of DEGs in macrophages between GBC and GBA. **(i)** Projection of myeloid checkpoints expression in macrophage subtypes on t-SNE plot. **(j)** Dotplot shows the cell communication between ERFs and each macrophage subtypes through collagen signaling. **(k)** Dotplot shows the gene marker expression of macrophage subtypes. .

We re-clustered monocytes/macrophages and identified eight distinct subsets (**Figure 4a**). Macro01 and Macro02 are enriched in GBA (**Figure 4b**). Macro01 highly expresses PGC, RGS1, and pro-inflammatory cytokines TNF, which is associated with M1 phenotype (17–19) (**Figure 4k**). Macro02(FCGBP^+^CX3CR1^+^C3^+^) was identified in two early GBCs as an immunosuppressive cell subpopulation (7). In our data, TREM2 is one of the markers of Macro02. TREM2 scavenges phospholipids and lipoproteins, aligning with the extensive upregulation of lipid metabolism genes in Macro2 (**Figure 4k**). Macro03, Macro04 and Macro06 are preferentially enriched in GBC (**Figure 4b**). Macro03 extensively expresses chemokine ligand (**Figure 4k**), which plays an important role in the recruitment of tumor-promoting immune cells in the TME. Macro04 was reported as a MDSC-like macrophage, an intermediate state during the monocyte maturation into macrophages in tumors (7). Macro06 highly expresses gene signatures associated with better prognosis (SELENOP and FOLR2) and an M2 marker (MRC1) (**Figure 4k** **) (** 20, 21). Macro05 occupies similar proportions across GBA and GBC and displays prominently active proliferation features (MKI67, STMN, TOP2A), likely serving as self-renewal gallbladder-resident macrophages (**Figure 4k**). Mono01 highly expresses GPX1 and mitochondrial genes (**Figure 4k**), indicating their important role in oxidative stress in the TME. Mono02 expresses multiple immunoglobulin heavy constant gamma proteins (**Figure 4k**). Considering that these monocytes highly express SPP1 (**Supplementary Figure S5e**) and SPP1^+^ Macrophages were reported to express immunoglobulin-related genes and monocyte marker VCAN, these cells might be a subset of SPP1^+^ Macrophages, a kind of tumor-promoting macrophage associated with unfavorable prognosis of patients (22).

To compare the functional differences of these cells, we used Ucell to calculate the gene set score. The ratio of M1 to M2 scores reflects the phenotypic tendency of different macrophage subsets. Using gallbladder-resident macrophages (Macro05) as a reference, Macro02 and Macro03 show an M2 inclination, while Macro01 and Macro06 share a similar profile to Macro05. The remaining cell subsets exhibit an M2 inclination (**Figure 4c**). In GBA, macrophages are predominantly of the M1 and intermediate phenotypes. In contrast, GBC contains diverse macrophage subtypes dominated by M2 (**Figure 4b**). Comparing the M1/M2 scores of all macrophages between GBA and GBC also confirms this (**Figure 4f**). However, the inflammatory and cytokine response is more active in GBC (**Figure 4g**). We performed GSEA enrichment analysis for DEGs of macrophages between GBC and GBA. The result revealed that the macrophages in GBC have enhanced chemotactic activity and downregulated antigen-presenting function compared with those in GBA (**Figure 4h**). The enhanced chemotaxis is mainly attributed to Macro03 (CCL2^+^) (**Figure 4d**). We also noticed that Macro04 (S100A9^+^) shows the lowest score in all gene sets with high variation (**Figures 4c, d**), suggesting the distinct maturity status of these cells. GO analysis reveals that Macro04 is associated with the regulation of immune response (**Figure 4e**). Just like T cells, macrophages also express immune checkpoints, and we visualized the expression of these molecules. The expression of SIRPA, LILRB1, LILRB2, Siglec1, Siglec7, Siglec9, PDCD1, CD163 and MARCO are preferentially expressed on Mono01, Mono02 and Macro03 (**Figure 4i**), suggesting their tumor-promoting roles. Besides, these cell subpopulations also interact with ERFs through CD44 (**Figure 4j**).

**Figure 5:**
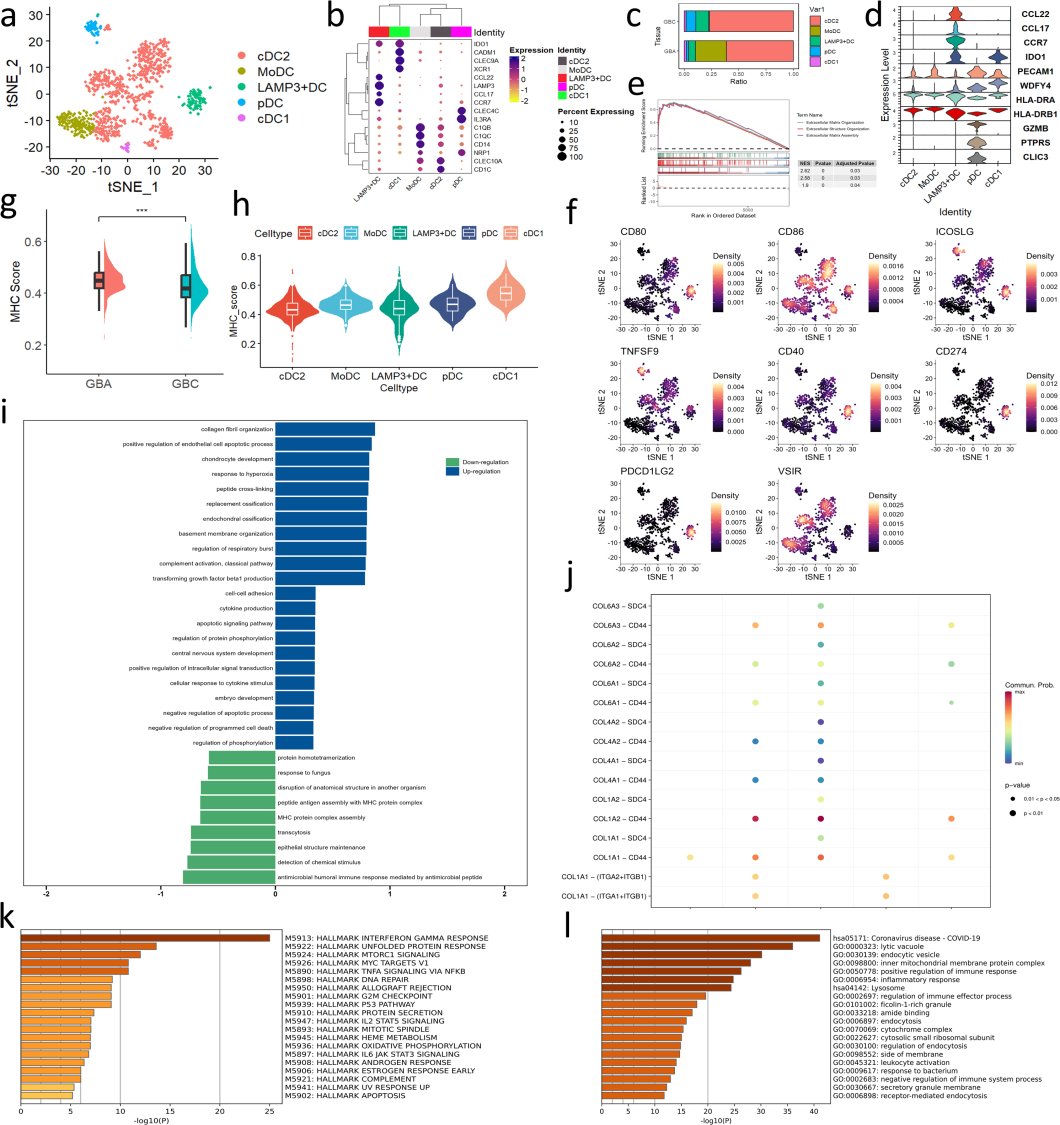
**(a)** t-SNE plot visualizing DC subsets from all samples. **(b)** Dotplot shows the gene marker expression of DC subtypes. **(c)** Bar charts showing the relative abundance of various macrophages subtypes in GBC and GBA. **(d)** Violin plot shows the gene signatures of DC subtypes. **(e, i)** GSEA enrichment analysis of DEGs in cDC2 between GBC and GBA. **(f)** Projection of costimulatory or coinhibitory molecules expression of DC subtypes on t-SNE plot. **(g)** MHC scores of all DCs in GBA and GBC. (h) MHC scores of each DC subtypes in GBA and GBC. **(j)** Dotplot shows the cell communication between ERFs and each DC subtypes through collagen signaling. **(k)** HALLMARK enrichment analysis of pDC. **(l)** GO and KEGG analysis of MoDC.

Dendritic cells (DCs) are composed of type 1 conventional DCs (cDC1s), type 2 cDCs (cDC2s) and plasmacytoid DCs (pDCs) (**Figures 5a, b**). We also identified LAMP3^+^DCs in our data. LAMP3^+^DC is a mature and migratory DC subset lacking the expression of cDC or pDC markers (**Figure 5b** **) (** 22). In our data, LAMP3^+^DCs are enriched in GBC with high expression of costimulatory or coinhibitory molecules, such as CD80, CD86, CD40, ICOSL, PD-1, and PD-L1 (**Figures 5c, f**). Therefore, the abundance of LAMP3^+^ DCs may serve as a potential indicator for predicting the efficacy of immunotherapy. LAMP3^+^DCs also express chemokines (CCL22, CCL17) and immunosuppressive IDO1, suggesting their great immunoregulatory capacity (**Figure 5d**). Similarly, pDCs enriched in GBC also exhibit immunosuppressive roles with high expression of GZMB, PTPRS, CLIC3, and a tolerogenic marker PECAM1 (CD31) (23) (**Figures 5c, d**). GZMB can inhibit the T cell proliferation (24), PTPRS can inhibit the production of interferon, and CLIC3 takes part in the angiogenesis and increases the invasiveness of cancer cells (25, 26). However, pDCs also highly express immunostimulatory molecules ICOSL and 4-1BBL (**Figure 5f**) and are enriched in the IFN-γ response (**Figure 5k**), suggesting their complex immunoregulatory activity. monocyte-derived dendritic cells (MoDCs) are enriched in GBA (**Figure 5c**). MoDCs mostly generate in response to inflammation and regulate the differentiation of CD4^+^T cells towards a type 1 T helper cell (Th1 cell), type 2 T helper cell (Th2 cell), or IL-17-producing T helper cell (Th17 cell) phenotype (27). GO and KEGG analysis reveals that MoDCs take part in the inflammation response and regulation of the immune system (**Figure 5l**). The cDC is the main subtype of DCs, playing an important role in antigen presentation. Both cDC1 and cDC2 highly express MHCII molecules, and cDC1 also express WDFY4 for cross-presentation (**Figure 5d** **) (** 28). To compare the antigen presentation capacity of different DC subtypes, we calculate the MHC score of each DC cell. To our surprise, cDC2, the main cell subtype of antigen presentation (27), exhibits a relatively low MHC score (**Figure 5h**). We suppose that the abnormal phenomena might be attributed to the education of TME in GBC. Consistent with our hypothesis, cDC2 in GBA exhibits a higher MHC score than GBC (**Figure 5g**). GSEA analysis shows that cDC2 in GBC upregulates the interaction with stroma components while downregulates MHC protein complex assembly (**Figure 5i**). Besides, the interaction between ERFs and cDC2 also presents a high communication probability through COL1A2-CD44 (**Figure 5j**). However, the mechanisms underlying the regulation of MHC assembly by ERFs require further elucidation. LAMP3^+^DCs also show strong interaction with ERFs through COL1A2-CD44 (**Figure 5j**), which supports our theory that ERFs facilitate the tissue retention of tumor-promoting immune cells.

### Identification of T and NK subsets that communicate with ERFs

**Figure 6:**
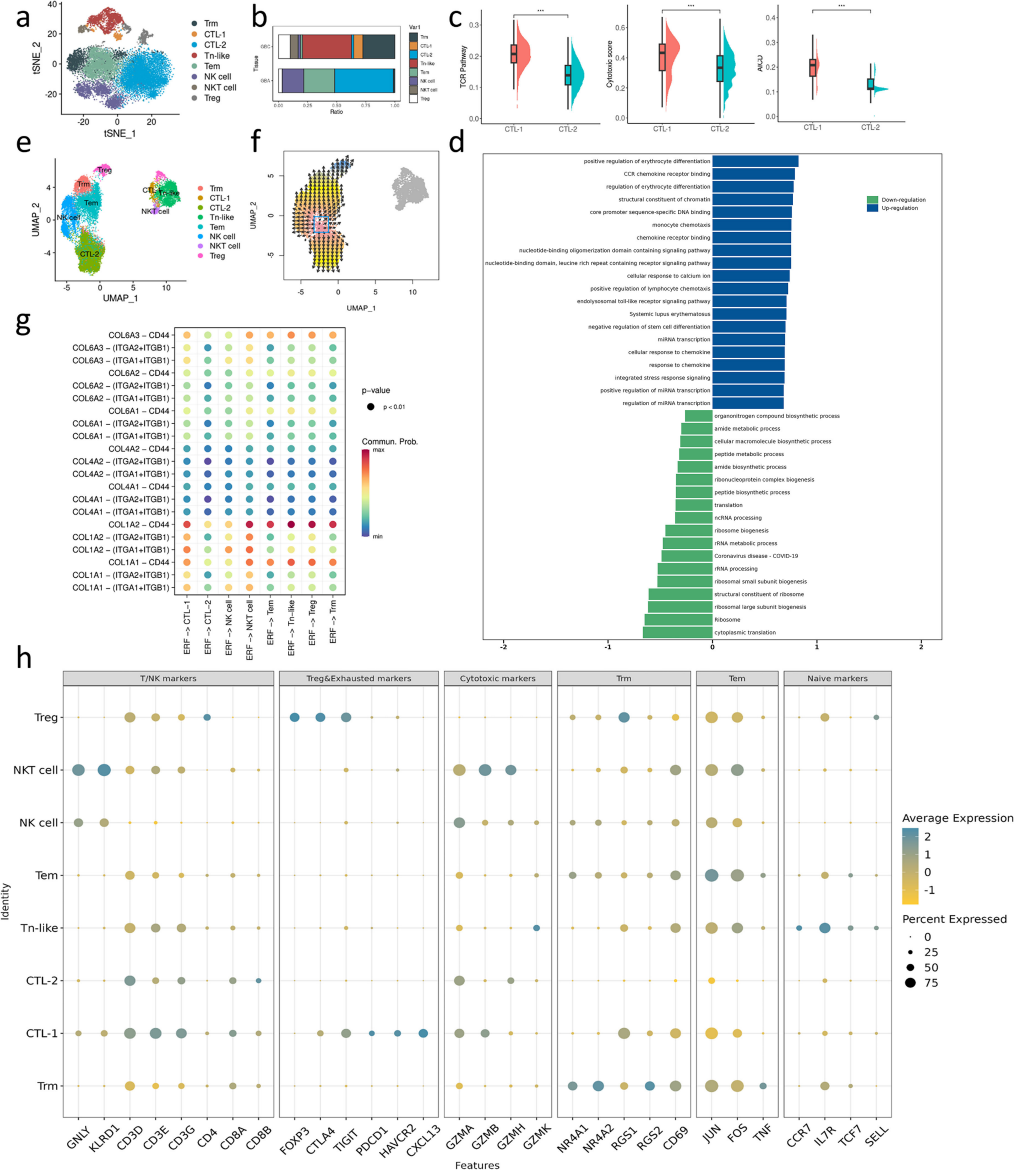
**(a)** t-SNE plot visualizing macrophage subsets from GBC and GBA. **(b)** Bar charts showing the relative abundance of various T/NK subtypes in GBC and GBA. **(c)** TCR, cytotoxic and AICD score between CTL-1 and CTL-2. **(d)** GSEA enrichment analysis of DEGs between CTL-1 and CTL-2 **(e)** umap plot visualizing macrophage subsets from all samples. **(f)** The direction of T cell subtypes differentiation. **(g)** Dotplot shows the cell communication between ERFs and each T/NK subtypes through collagen signaling. **(h)** Dotplot shows the gene marker expression of T/NK subtypes.

By clustering T/NK cells, we identified eight subsets in GBC and GBA. T/NK subsets show different distributions between GBC and GBA (**Figure 6a**). Regulatory T cells (Tregs), NKT cells, Naïve-like T (Tn-like) cells, cytotoxic T cell (CTL)-1, and tissue-resident memory T cells (Trms) are enriched in GBC (**Figure 6b**), while NK cell, effector memory T cells (Tems) and CTL-2 are enriched in GBA (**Figure 6b**). Both CTL-1 and CTL-2 highly express cytotoxic granzyme (**Figure 6h**). CTL-1 mainly expresses GZMA and GZMB (**Figure 6h**), while CTL-2 mainly expresses GZMA and GZMH (**Figure 6h**). Compared with CTL-2, CTL-1 presents more activated TCR signaling and higher cytotoxic ability (**Figure 6c**). However, CTL-1 also exhibits an activation-induced cell death (AICD) (**Figure 6c**) with high expression of exhausted markers including CTLA4, TIGIT, PDCD1, and CXCL13 (**Figure 6h**). GSEA analysis reveals that CTL-1 has a stronger chemotaxis ability, and the terms associated with the biosynthesis of ribosomes are enriched in CTL-2 (**Figure 6d**). These results indicate that CTL-1 in GBC represents a more mature and exhausted phenotype compared with CTL-2 in GBA. Trms highly express tissue resident markers NR4A1, NR4A2, RGS1, RGS2 and CD69 (**Figure 6h**). The NR4A nuclear receptor family was identified as a key mediator of T cell exhaustion (29, 30), and RGS1 can impede the T cell infiltration into tumors and inhibit their cytotoxic capacity (31). Tems in our data are characterized by the expression of JUN, FOS, TNF, and CD69 (**Figure 6h**), which is consistent with the cell subset identified in hepatic sinuses by Huang. et al (32). We used VECTOR to infer the developmental directions of T cell subtypes. The results show that Tems have the potential to differentiate into Trms (**Figures 6e, f**). Tn-like cells highly express GZMK besides naïve markers. Previous study suggests that GZMK^+^T cells are a precursor of exhausted T cells (22). The proximity in the UMAP and t-SNE between Tn-like cells and CTL-1 with exhausted markers also suggests their similarity in gene signatures (**Figures 6a, e**).

Cellchat analysis reveals that CTL-1, NKT cells, Tems, Tn-like cells, Treg and Trms can interact with ERFs through COL1A2-CD44 (**Figure 6g**). Most of them show preferential accumulation in GBC and have an immunosuppressive or exhausted phenotype. The results support the important role of ERFs in the formation and maintenance of the tumor immune microenvironment (TIME).

## Conclusion and discussion

Metaplasia-dysplasia-carcinoma and adenoma-carcinoma are well-recognized pathways from precancerous lesions to invasive GBC. Compared with the metaplasia-dysplasia-carcinoma sequence, the adenoma-carcinoma sequence is poorly characterized (3). The scarcity of clinical cases, as well as a lack of proper GBA cell lines and animal models, presents significant challenges to research in this field. In the past few years, scRNA-seq technologies have shed light on the mechanism of adenoma-carcinoma sequence from the perspective of TME. TME is a complex ecosystem containing cancer cells surrounded by various non-malignant cells, collectively embedded in an altered, vascularized ECM, which plays an important role in the initiation, progression, and metastasis of cancer (5). Previous studies revealed the evolutionary similarity and compared the composition of immune cells between GBA and GBC (13). Our study suggests that EMT mediates the malignant transformation of epithelial cells in GBA and CAFs dominate the difference in immune cell composition between GBC and GBA. The tumor-specific CAF called ERF is characterized by high expression of collagen and acts in the ECM remodeling of GBC. Consistent with previous studies, IRFs can differentiate into ERFs, and both of them proceed towards SLFs. However, both IRFs and ERFs are absent in the GBA of our data. The origin of CAFs during GBA malignant transformation requires further elucidation.

EMT plays an important role in the initiation, invasion and metastasis of tumors. Wnt/β-catenin signaling can induce EMT in various types of cancer (33). The mutation and high expression of β-catenin in GBA cells have been widely recognized (34). Lin et al. performed laser microdissections on tissue sections to isolate tissues of normal gallbladder tissues, BilIN, and GBC from the same individuals, and identified the critical roles of CTNNB1 mutation in the tumorigenesis of gallbladder (35). However, frequent CTNNB1 mutations and rare malignant transformation in GBA seem to be contradictory. In the mouse model of breast cancer, SDC1 is required for Wnt-induced carcinogenesis without the influence on Wnt or β-catenin expression (15, 16, 36). In our study, ERFs exhibit a strong interaction with epithelial cells through COL1A2-SDC1. Besides, SDC1^+^ cells exhibit a higher EMT score than SDC1^-^ cells among CTNNB1^+^epithelial cells in GBA (**Supplementary Figure S6f**). Considering the wide expression of SDC1 in GBA (**Supplementary Figures S6a, b**), ERFs can potentially activate Wnt-β-catenin induced GBA-GBC transformation through COL1A2-SDC1.

Immune cells can be either tumor-suppressive or tumor-promoting in TME. The difference between the inflammatory microenvironment that induces tumorigenesis and immunosuppressive TME is an interesting topic. Our study revealed that macrophages in GBC exhibit an active M2 phenotype with enhanced chemotaxis, while macrophages in GBA are mainly M1 phenotype. LAMP3^+^DCs and pDCs with immunosuppressive roles are enriched in GBC, while MoDCs in response to inflammation show preferential distribution in GBA. Besides, cDC2s, the main DC subtype for antigen presentation, exhibit relatively low MHC molecule expression compared with those in GBA. There are obvious differences in T cell subtypes between GBC and GBA. In GBC, immunosuppressive Tregs and exhausted/pre-exhausted T cells occupy a relatively high proportion, such as CTL-1, Trm, and Tn-like cells in our data. In GBA, CTL-2 exhibits a relatively immature phenotype. Both T cells in GBA and GBC have a dysfunctional state. He. et al. also revealed the accumulation of Treg and exhausted T cells in GBC. Besides, follicular helper T cells (Tfhs) are also enriched in GBC compared with GBA (13). However, due to the limited sample size and inevitable cell loss during tissue dissociation procedures, as well as in the quality control of scRNA-seq data, our findings merely reveal a portion of the disparities in the TIME between GBC and GBA. We anticipate that collaborative multicenter studies and the continuous advancement of sequencing technologies in the future will lead to significant breakthroughs in addressing this issue.

Fibroblasts are principal components of stroma cells and play an important role in tissue repair and regeneration after injury (37). The persistent stimulation from cancer can irreversibly drive fibroblasts to acquire a cancer-associated phenotype (38). One major feature of CAFs is their ability to produce large amounts of ECM proteins, such as collagens, glycoproteins, and proteoglycans (38). Collagen, the most abundant component of the ECM, is associated with lower survival rates in cancer patients (39). Our data also shows that ERFs expressing high levels of collagen are found in GBC samples. In addition to collagen, other proteins secreted by CAFs, such as fibronectin, are also important in tumor progression. However, the role of fibronectin in cancer seems to be complex, as fibronectin in the TME or endogenously expressed in tumor cells can have opposite effects on patient prognosis (39). The activation of CAF secretion patterns contributes to ECM remodeling in tumors and thus is involved in tumor invasion, metastasis, and immune responses (38, 40, 41). ERFs mainly interact with immune cells through COL1A2-CD44. CD44 is a kind of glycoprotein involved in cell adhesion. CAF-mediated ECM remodeling provides a wide docking site for immune cells with expression of CD44. In colorectal tissue, adenomas and carcinomas share similar stromal features (42). An immunosuppressive microenvironment is established early in the adenoma stage, which may explain the higher risk of malignant transformation in familial adenomatous polyposis (FAP) (43). Myofibroblasts in FAP can secrete CXCL14, driving Treg differentiation (43). Additionally, fibroblast-secreted collagen interacts with CD44 on TAMs, increasing their PD-L1 expression (44). However, the effects of collagen-CD44 interactions on immune cell phenotypes in gallbladder cancer require further investigation. The similar expression between GBC and para-carcinoma tissue supports that CD44 expression on immune cells (**Supplementary Figures S6a, c-e**) is not induced by TME, and CD44 might contribute to the migration of immune cells. These results suggest that ERF may serve as a potential biomarker for early diagnosis and immunotherapy sensitivity in GBC.

In this study, we compared the cell compositions of TME between GBC and GBA. The results revealed that EMT plays an important role in the malignant transformation of GBA cells, and ERFs govern the immune cell difference through COL1A2-CD44. This study paves the way for further research on the underlying mechanism of the adenoma-carcinoma sequence and the malignant biological behaviors of GBC.

## Limitations

The small sample size (n=4 per group) limits the representativeness of the conclusion and the detection of rare cell populations in GBC and GBA, partly due to the rarity of these diseases and challenges in sample collection. Our analysis also focuses solely on the transcriptional level of collagen-mediated cell communication. Larger studies integrating protein-level analysis and cell-cell communication disruption are needed to validate and expand upon our findings.

## Data Availability

The raw data supporting the conclusions of this article will be made available by the authors, without undue reservation.
