# Supplementary figures and images for: Extracellular matrix remodeling fibroblasts govern the tumor microenvironment disparity between adenomatous lesions and adenocarcinoma in gallbladder
Source: Front Immunol. 2025 Jul 18;16:1637300. doi: 10.3389/fimmu.2025.1637300 (PMC12313498; doi:10.3389/fimmu.2025.1637300)

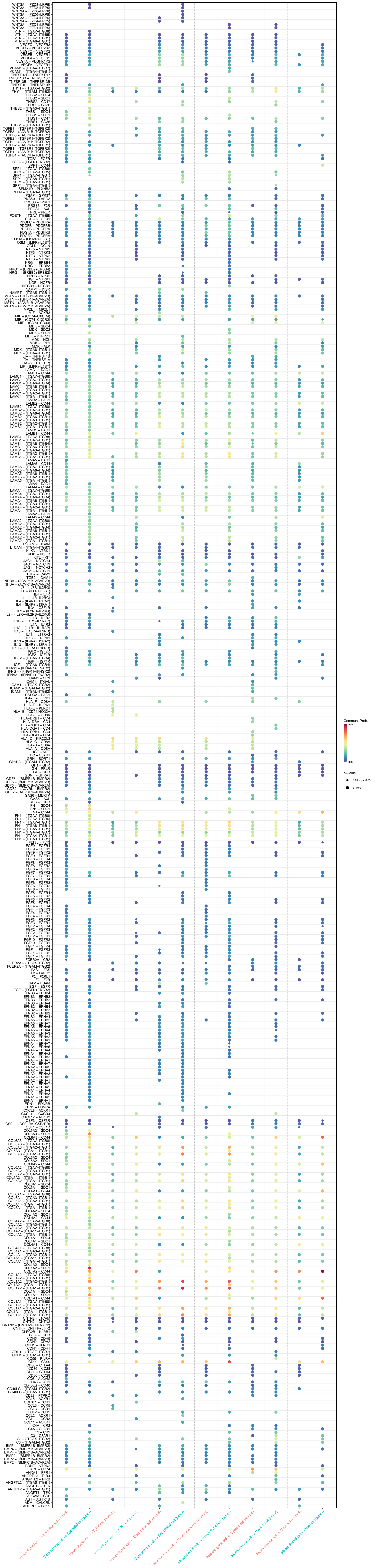

Supplement: Supplementary file 1 [file DataSheet1.pdf]
