# Supplementary material for: Extracellular matrix remodeling fibroblasts govern the tumor microenvironment disparity between adenomatous lesions and adenocarcinoma in gallbladder
Source: Front Immunol. 2025 Jul 18;16:1637300. doi: 10.3389/fimmu.2025.1637300 (PMC12313498; doi:10.3389/fimmu.2025.1637300)

**Patients Information**

| Patients ID | Sex | Age | Disease | Stage |
| --- | --- | --- | --- | --- |
| GBAC1 | female | 68 | Gallbladder adenocarcinoma | Ⅱb(T2bN0M0) |
| GBAC2 | female | 68 |  | Ⅰ(T1bN0M0) |
| GBAC3 | male | 68 |  | Ⅱa(T2bN0M0) |
| GBAC4 | female | 71 |  | Ⅲb(T3N1M0) |
| GBA1_S1 | male | 40 | ICPN | - |
| GBA1_S2 |  |  |  |  |
| GBA2 | male | 75 |  |  |
| GBA3 | male | 77 |  |  |


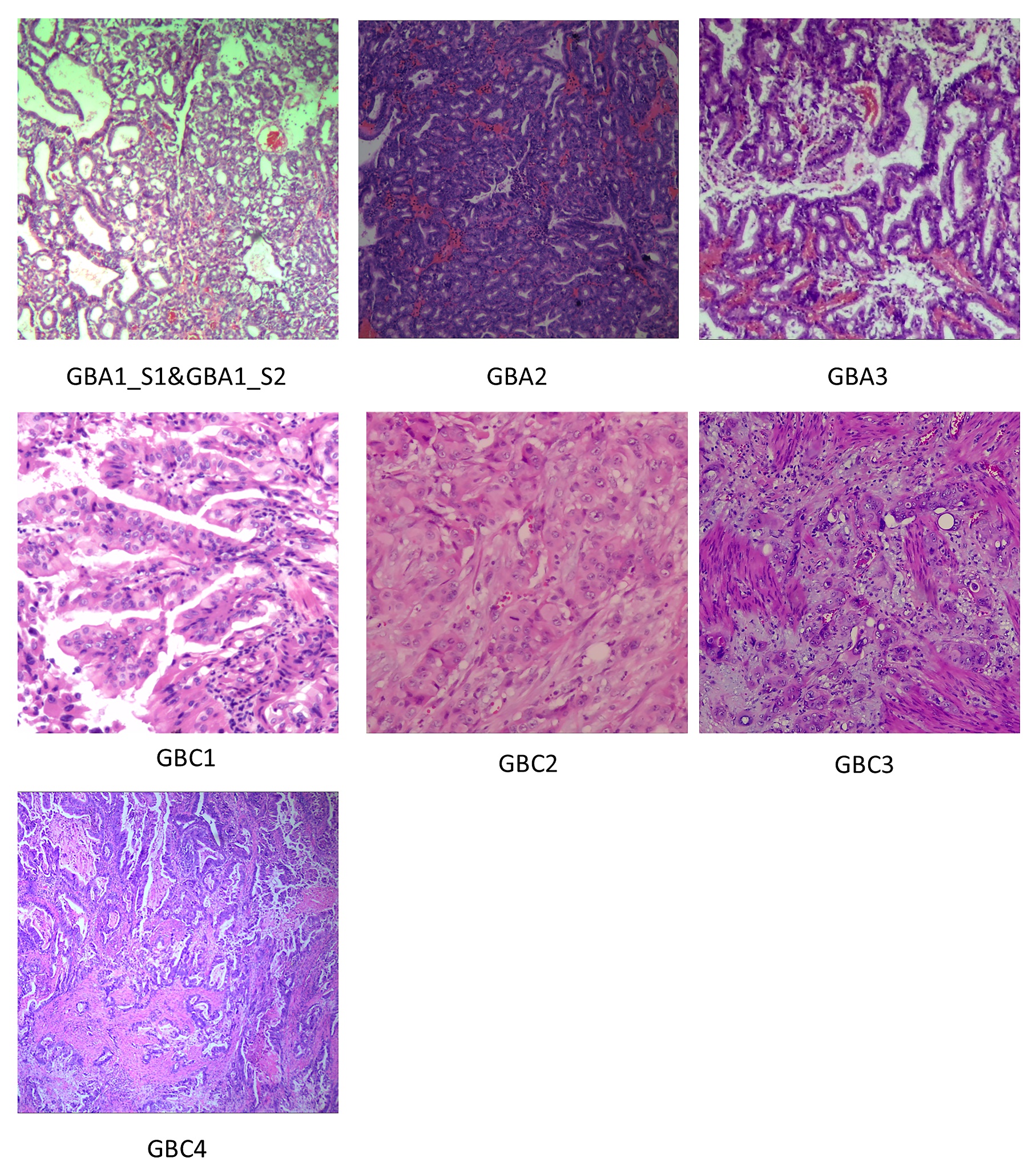

Supplement: Supplementary file 2 [file Table1.docx]
