# Supplementary material for: Extracellular matrix remodeling fibroblasts govern the tumor microenvironment disparity between adenomatous lesions and adenocarcinoma in gallbladder
Source: Front Immunol. 2025 Jul 18;16:1637300. doi: 10.3389/fimmu.2025.1637300 (PMC12313498; doi:10.3389/fimmu.2025.1637300)
Supplement: Supplementary file 3 [file Table2.docx]

**Figure S1.**Dotplot plot visualizing expression levels of cell-type gene signatures among identified cell types.


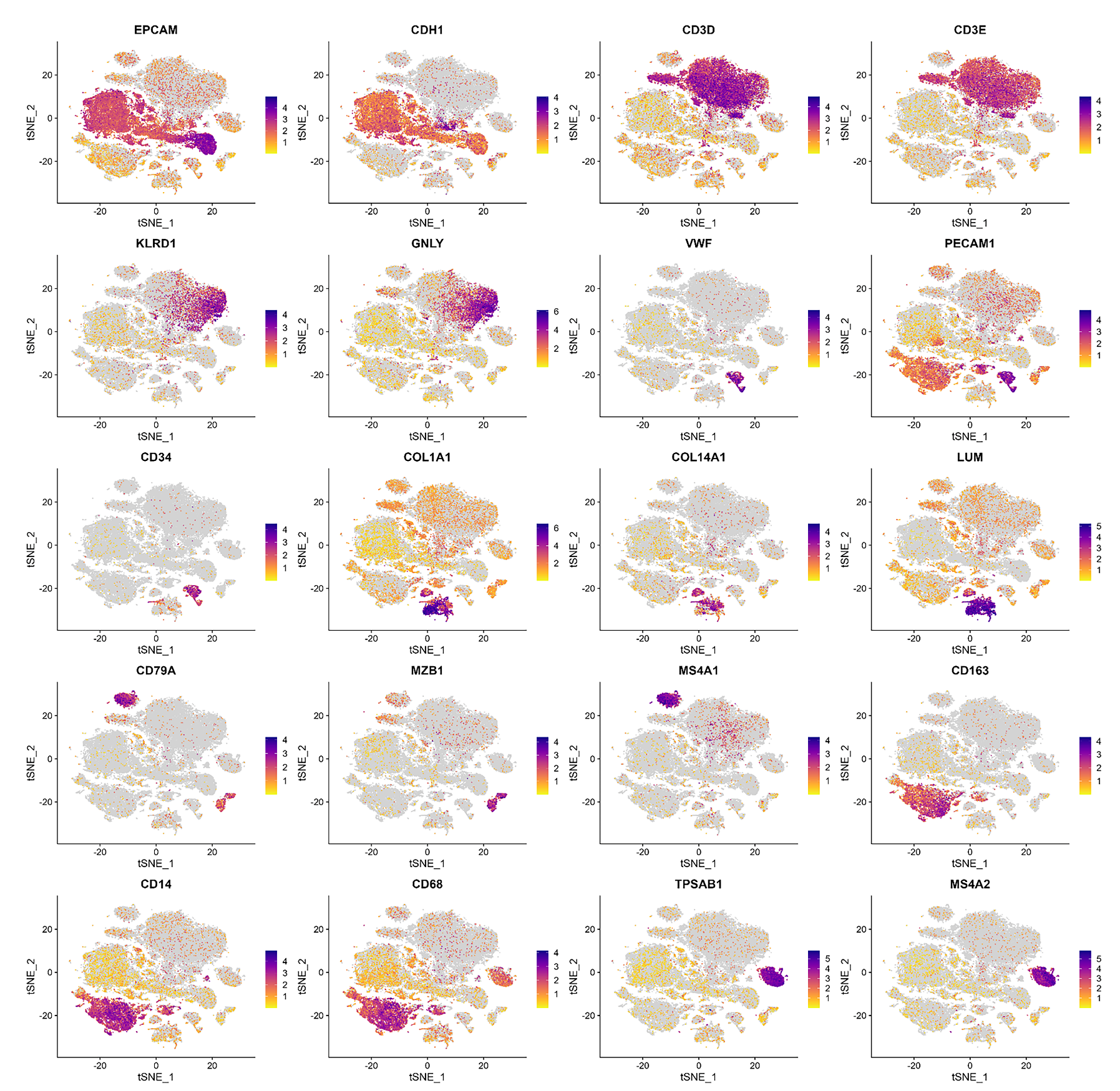


**
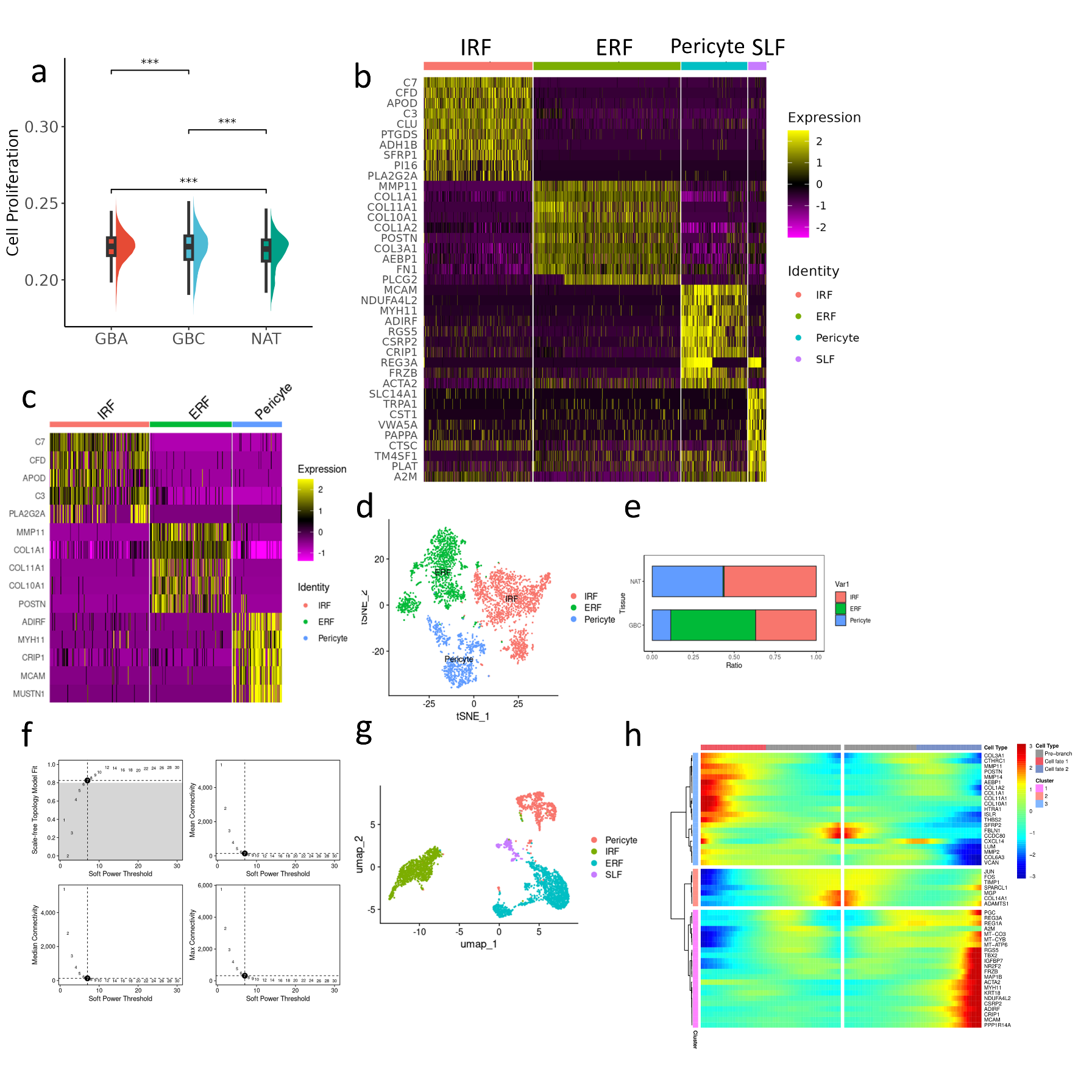
Figure S2 a.** Cell proliferation score of GBA, GBC and adjacent normal tissue of GBC. **b.** Heatmap for top 10 gene markers of mesenchymal subtypes in GBC and GBA. **c.** Heatmap for top 5 gene markers of mesenchymal subtypes in GBC and adjacent normal tissue. **d.** t-SNE plot visualizing mesenchymal subsets from GBC and adjacent normal tissue. **e.** Bar charts showing the relative abundance of various mesenchymal subtypes in GBC and adjacent normal tissue. **f.** soft power threshold selection for hdWGCNA of ERFs. **g.** umap plot visualizing mesenchymal subsets from GBC and GBA. **h.** BEAM genes of mesenchymal cell subtypes trajectory.


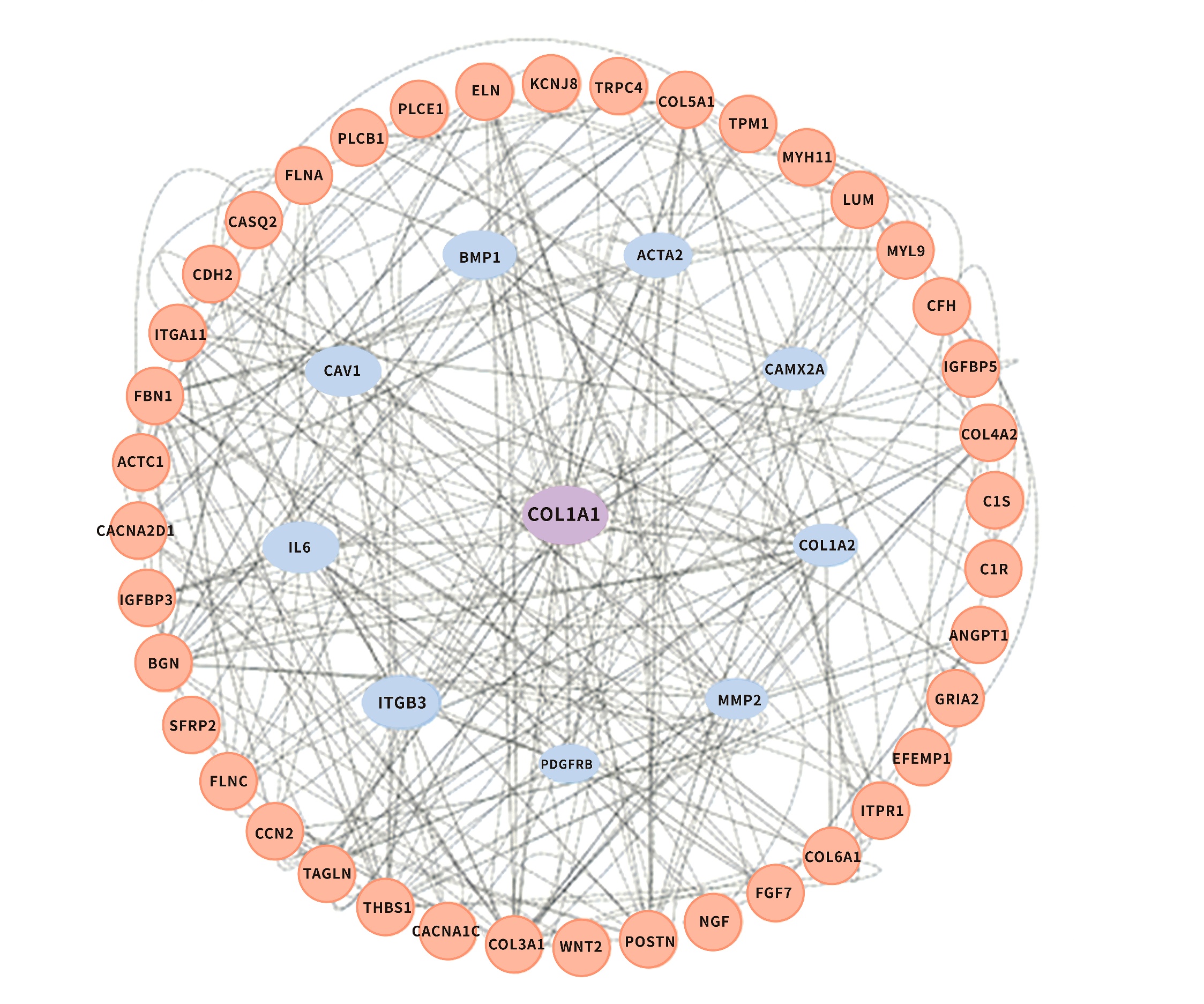


**Figure S3.** PPI for DEGs with top50 BC value between GBC and GBA in GSE202479.


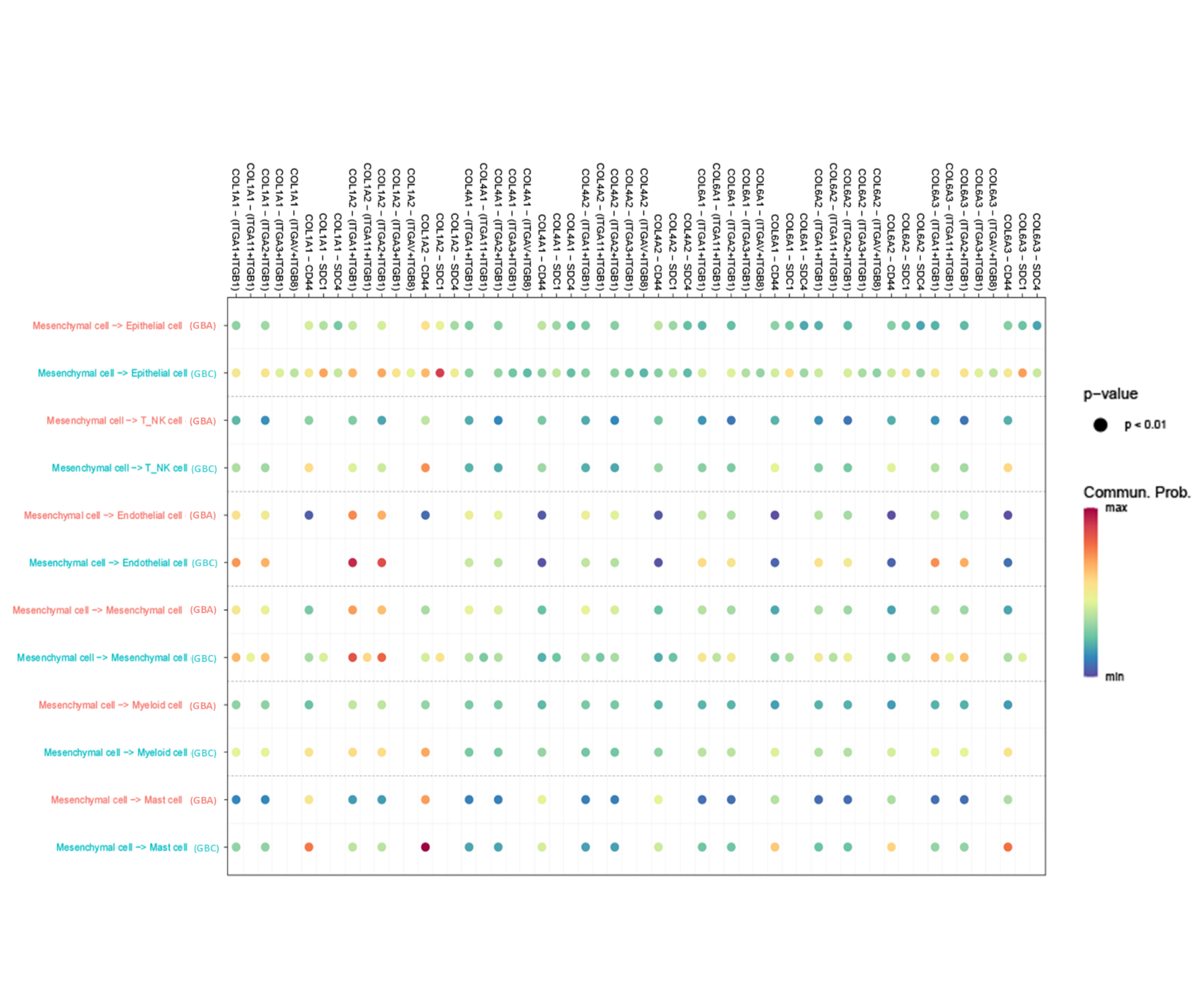


**Figure S4**.Dotplot shows the differential cell communication between mesenchymal cells and other cell types through collagen signaling.


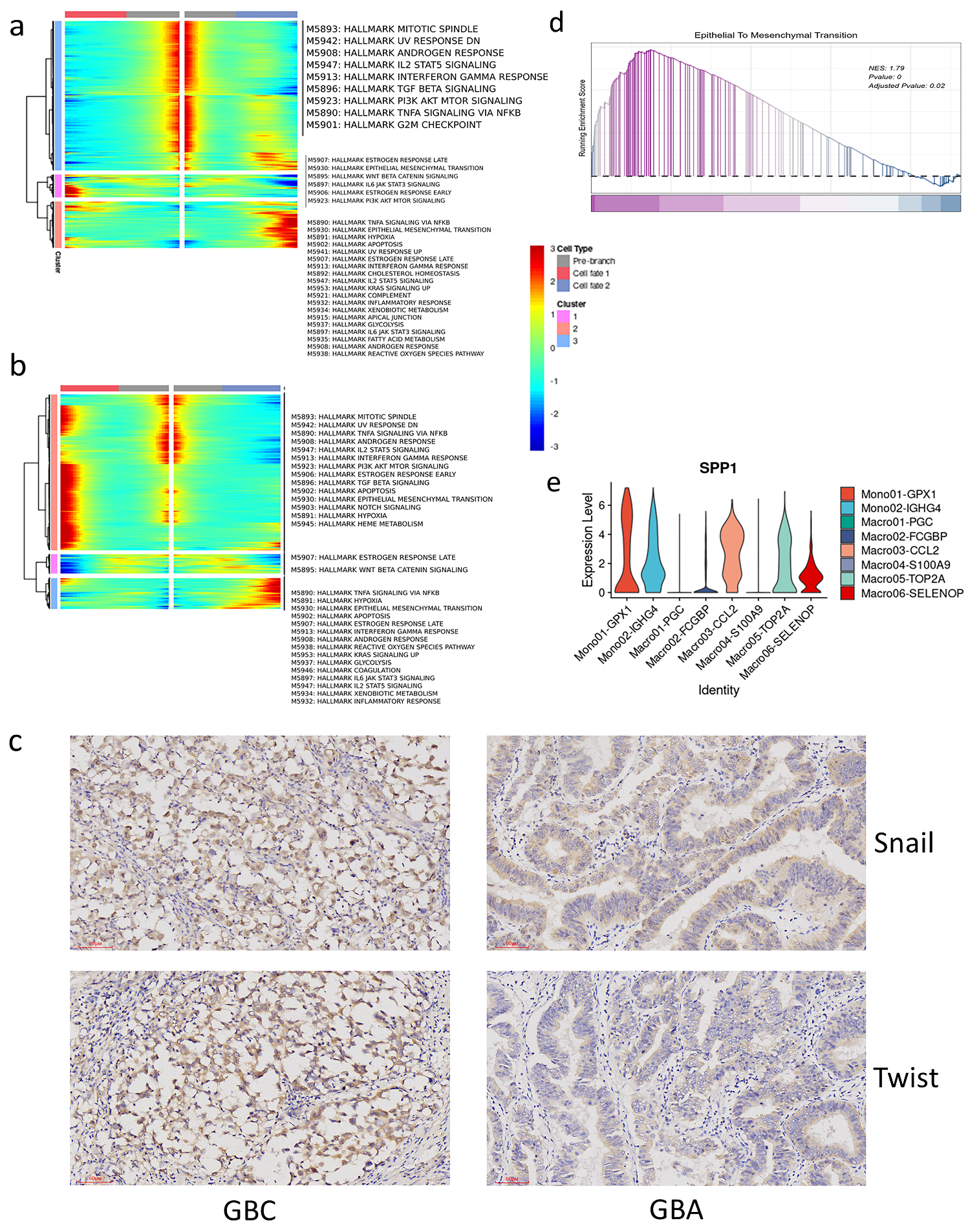


**Figure S5 a**. Enrichment analysis of BEAM genes in epithelial cells trajectory. (branch point 1) **b.** Enrichment analysis of BEAM genes in epithelial cells trajectory. (branch point 2) **c.** EMT markers (Snail and Twist) expression in GBC and GBA. **d.**GSEA enrichment analysis of DEGs between early GBC and GBA in GSE202479 datasets. **e.** Violin plot shows SPP1 expression on different subtypes of macrophages


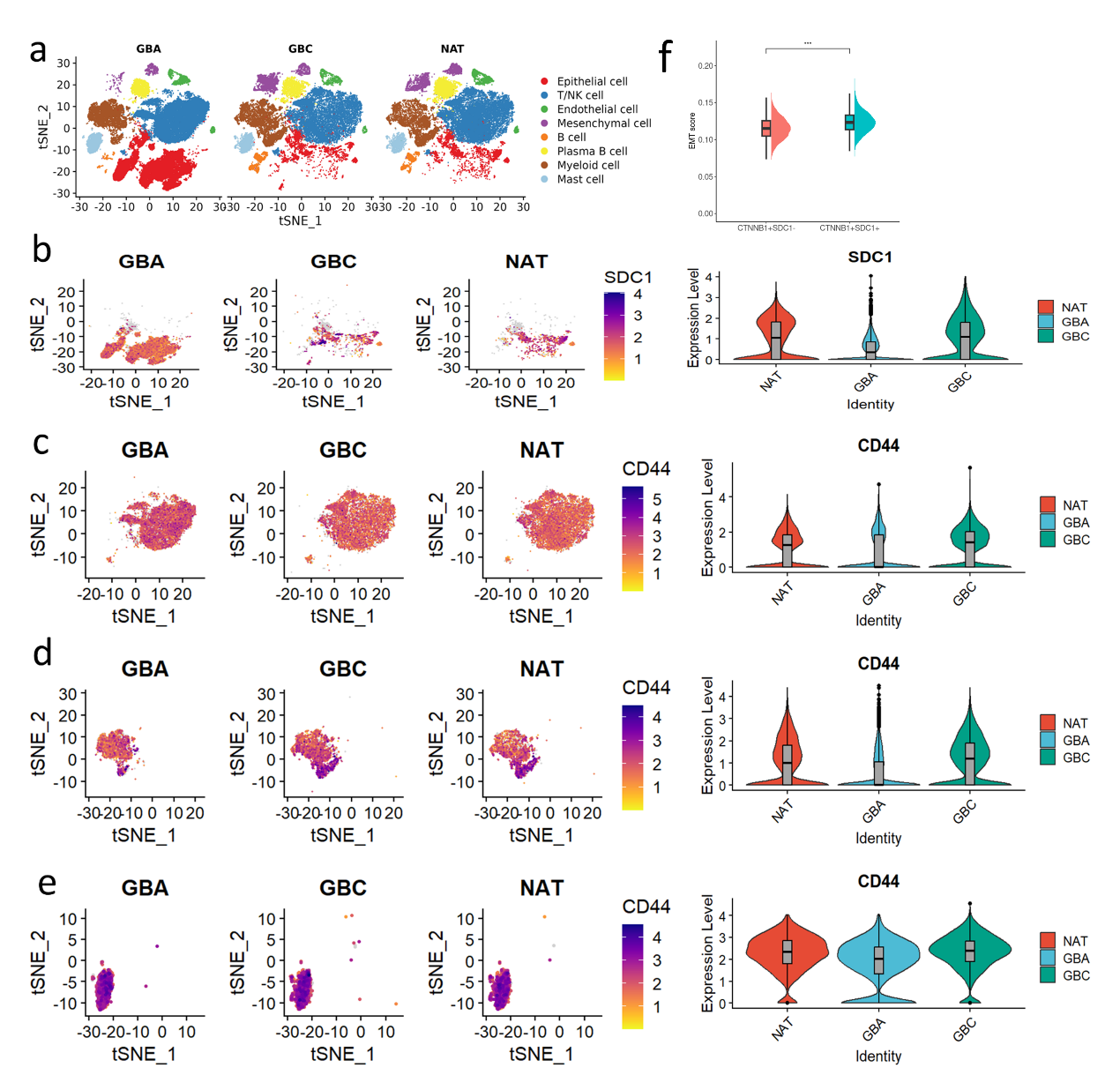


**Figure S6 a**. t-SNE plot visualizing cells from all samples splited by tissue type. **b.** SDC1 expression in epithelial cells from GBA, GBC and adjacent normal tissue of GBC. **c.** CD44 expression in T/NK cells from GBA, GBC and adjacent normal tissue of GBC. **d.** CD44 expression in myeloid cells from GBA, GBC and adjacent normal tissue of GBC. **e.** CD44 expression in mast cells from GBA, GBC and adjacent normal tissue of GBC. **f.** EMT score of CTNNB1+SDC1+ and CTNNB1+SDC1- cells in epithelial cells of GBA
